# Severe Hyperthyroidism Complicated by Agranulocytosis Treated with Therapeutic Plasma Exchange: Case Report and Review of the Literature

**DOI:** 10.1155/2018/4135940

**Published:** 2018-01-10

**Authors:** Vishnu Garla, Karthik Kovvuru, Shradha Ahuja, Venkatataman Palabindala, Bharat Malhotra, Sohail Abdul Salim

**Affiliations:** Department of Internal Medicine, University of Mississippi Medical Center, Jackson, MS, USA

## Abstract

**Aim:**

To present a case of Graves' disease complicated by methimazole induced agranulocytosis treated with therapeutic plasma exchange (TPE) and review of the literature.

**Case Presentation:**

A 21-year-old patient with a history of Graves' disease presented to the endocrine clinic. His history was significant for heat intolerance, weight loss, and tremors. Upon examination he had tachycardia, smooth goiter, thyroid bruit, and hyperactive reflexes. He was started on methimazole and metoprolol and thyroidectomy was to be done once his thyroid function tests normalized. On follow-up, the patient symptoms persisted. Complete blood count done showed a white blood cell count of 2100 (4000–11,000 cells/cu mm) with a neutrophil count of 400 cells/cu mm, consistent with neutropenia. He was admitted to the hospital and underwent 3 cycles of TPE and was also given filgrastim. He improved clinically and his thyroxine (T4) levels also came down. Thyroidectomy was done. He was discharged on levothyroxine for postsurgical hypothyroidism.

**Conclusion:**

Plasmapheresis may be useful in the treatment of hyperthyroidism. It works by removing protein bound hormones and also possibly inflammatory cytokines. Further studies are needed to clarify the role of various modalities of TPE in the treatment of hyperthyroidism.

## 1. Introduction

Hyperthyroidism is an overproduction and persistent release of thyroid hormones, while thyrotoxicosis refers to the set of clinical manifestations secondary to excessive thyroid hormone action on the tissues [1]. Conventionally thyrotoxicosis is treated medically using agents which inhibit the synthesis and release of thyroid hormones [2]. TPE was first used as a modality in the treatment of hyperthyroidism in the 1970s; however, till this date the role of TPE in the treatment of hyperthyroidism is unclear [3, 4].

We present a case of Graves' disease complicated by agranulocytosis treated with TPE along with a pertinent review of the literature.

## 2. Case Description

A 21-year-old male patient presented to the emergency department with neck pain and dysphagia. He had been diagnosed with Graves' disease about 4 years ago; however, he was not taking any medication for the last 2 years. Upon further enquiry, the patient admitted to a history of weight loss, palpitations, tremors, and lack of sleep. Vital signs showed a heart rate of 130/minute, blood pressure of 132/67 mm Hg, respiratory rate of 18/minute, and temperature of 97.8. Examination revealed an anxious patient with bilateral lid lag, large smooth goiter with a thyroid bruit, and tremors of upper extremities. Laboratory assessment revealed a suppressed TSH, high free t4, free t3, positive antithyrotropin receptor antibodies (TRab), and thyroid stimulating immunoglobulin (TSI) confirming the diagnosis of Graves' disease (Table 1). Ultrasound of the neck showed an enlarged hypervascular thyroid gland consistent with Graves' disease. Methimazole and atenolol were started. Thyroidectomy was planned to be done once the thyroid function tests normalized. The patient was discharged from the hospital and was to follow up in the endocrine clinic in 1 month. Upon follow-up in the endocrine clinic, the patient admitted that he had been noncompliant with his medications for a week. He also complained of heat intolerance, weight loss, insomnia, palpitations, and a sore throat. Again noted on exam were tachycardia, a smooth goiter with bruit, tremors, and hyperactive reflexes in all extremities. TSH was suppressed, free t4 and total t3 were high, and complete blood count showed a low white blood cell count (WBC) and low absolute neutrophil count. A diagnosis of methimazole induced agranulocytosis was made and the patient was admitted to the hospital.

Hematology was consulted for TPE to control hyperthyroidism and also administration of filgrastim for neutropenia. Three treatments of plasma exchanges were done 2 days apart. The replacement fluid used was half albumin and half plasma. Filgrastim was administered daily. WBC and neutrophil counts improved significantly and normalized. Patient continued to improve clinically and his free t4, previously in the unmeasurable range, did come down. Thyroidectomy was done and pathology revealed an enlarged thyroid with diffuse hyperplasia. Postoperatively, he developed hypocalcemia and was treated with calcium carbonate. Levothyroxine was started for the treatment of postsurgical hypothyroidism. Upon follow-up, a month later in the endocrine clinic, the patient was doing well on levothyroxine.

## 3. Methods and Results

We searched PubMed using the following key words: hyperthyroidism and plasmapheresis. We restricted our search to publications in “English” and involving “human subjects.” Abstract of meetings and unpublished results were not included in our study. The last search was done on 6/27/2017.

The initial search resulted in 91 articles; 64 articles were excluded based on the title and abstract. Eligibility criteria were those articles which used TPE to treat hyperthyroidism. 27 articles met the inclusion criteria and were included (Table 2) [4–30].

## 4. Discussion

Thyroxine (T4) has the highest concentration among iodothyronines in the plasma and is produced exclusively by the thyroid; triiodothyronine (T3) is primarily derived (about 80%) from the peripheral tissues by deiodination of T4. T4 is about 68% bound to thyroxine binding globulin (TBG), 11% to transthyretin, and 20% to albumin. T3 is 80% bound to TBG, 9% to transthyretin, and 11% to albumin [1]. This extensive protein binding aids in the clearance of thyroid hormones during therapeutic plasma exchange (TPE) [31].

TPE is an extracorporeal blood purification technique used to for eliminating large molecular substances from the plasma [30]. In contrast to dialysis which cannot clear protein bound substances, TPE can clear protein bound substances [13]. The process involves passing the patient's blood through a medical device and separating the plasma out; it is then replaced with a colloid (albumin or plasma) or a combination of crystalloid and colloid. TPE clears thyroid hormones which are protein bound; the colloid used to replace the plasma provides new binding sites for thyroid hormone which are cleared during the next TPE session [6]. Besides thyroid hormones, TPE may help in the clearance of cytokines, deiodinase enzyme, and Graves' antibodies which help not only in the resolution of thyrotoxicosis but also of Graves' ophthalmopathy and pretibial myxedema [31].

There are a number of replacement fluids available, plasma as a replacement fluid offers the advantage of not depleting coagulation factors and also replenishing thyroxine binding globulin [20]. Human albumin offers the advantage of having a larger pool of low affinity binding sites for thyroid hormone [9]. We recommend plasma as the replacement fluid in patients with coagulation disorders or those who are going for surgery.

TPE was first used for the treatment of hyperthyroidism in 1970 by Ashkar et al. on 3 cases of thyroid storm [4]. Our literature review showed that TPE was used in 16 cases not responding to standard treatment, 13 cases of agranulocytosis or other side effects of thionamides, 8 cases of amiodarone induced thyrotoxicosis, and 5 cases for preparation of thyroidectomy. Petry et al. used TPE for the treatment of thyroid storm in postsleeve pneumonectomy patient who did not respond to the conventional treatment and thyroidectomy was considered high risk [22]. Jha et al. reported a case of thyroid storm secondary to excessive consumption thyroid supplements successfully treated with TPE. TPE was particularly useful as the patient had been taking excessive supplements for six days making the use of gastric decontamination and cholestyramine less useful [30].

Lew et al. used double filtration plasmapheresis (DFPP) in a patient with Graves' disease who needed surgical debridement. DFPP is a process where the plasma is first separated from the blood and then large molecules like immunoglobulins and lipoproteins are removed. The advantage would be lesser removal of coagulation factors making it useful in a patient who has to undergo surgery; however, small molecules may not be removed effectively by this procedure [11]. Koball et al. used a single pass albumin dialysis (SPAD) in a patient who had no clinical improvement after two sessions of plasmapheresis. Albumin dialysis has been used to eliminate toxins which accumulate in liver failure. The authors hypothesized that since this was a continuous procedure it would be effective in removing a greater quantity of hormone from the blood. It was also noted that if the plasmapheresis was followed by SPAD it decreased the chance of rebound increase of thyroid hormones [13].

The American society of apheresis categorizes the use of TPE in the treatment of hyperthyroidism as category III which states that the role of TPE has not been established in the treatment of thyroid storm. The recommended frequency of treatment is daily to once in three days till clinical improvement is noted [3].

TPE in the treatment of hyperthyroidism can be used when conventional treatment is not working or contraindicated. As noted in our literature review, it can be used in a variety of scenarios with clinical and biochemical improvement. Limitations of TPE include lack of wide spread availability, potential for hemodynamic instability, and the risk of infections.

## 5. Conclusions

In summary, TPE is a useful adjunct in the treatment of hyperthyroidism; its use is suggested in cases with severe thyrotoxicosis with cardiac or neurological complications, or when standard antithyroid treatments are either unresponsive or contraindicated. It is also a useful adjunct in treating cases with levothyroxine overdose. TPE should be done daily till clinical improvement is noted. Thyroid hormone status is monitored by checking free t4 and free t3 before and after every TPE session; however, clinical and biochemical dissociation may exist. More research is needed into the usefulness of DFPP and SPAD in the treatment of hyperthyroidism.

## Figures and Tables

**Table 1:** Laboratory assessment on admission.

TSH (0.27–4.2 mcIu/ml)	<0.01
Free t4 (0.9–1.7 ng/dl)	>7.77
Free t3 (0.8–2.0 ng/ml)	>6.51
WBC (4000–11,000 cells/cu mm)	2.1
Absolute neutrophil count	0.4
TRab (0–1.75 IU/L)	26
TSI (0–1.3)	5.5

**Table 2:** Literature review.

Authors	Cases	Indication	Indication for plasmapheresis	Outcome
Kaderli et al.	3	Amiodarone induced thyrotoxicosis	Amiodarone induced thyrotoxicosis	Underwent thyroidectomy

Min et al.	1	Graves' disease	Elevated liver function tests	Biochemical improvement with about 40% decrease in total T3

Aydemir et al.	1	Graves' disease	Jaundice	Biochemical improvement with greater than 60% decrease in FT4 and FT3

Bilir et al.	1	Graves' disease	Drug induced angioneurotic edema	Underwent thyroidectomy

Carhill et al.	2	Graves' disease	(1) Increase in transaminases (2) Unresponsive to standard treatment	Clinical and biochemical improvement

Vyas et al.	1	Exogenous intoxication	Exogenous etiology	Clinical and biochemical improvement

Lew et al.	1	Graves' disease	Agranulocytosis and hemophagocytosis	Clinical and biochemical improvement with greater than 80% decrease in FT4 and FT3

Enghofer et al.	1	Graves' disease	Fulminant hepatitis	Underwent thyroidectomy

Koball et al.	1	Unknown	Preparation for urgent thyroidectomy	Clinical and biochemical improvement

Ezer et al.	11	(7) Graves' disease (3) Toxic multinodular goiter (1) Iodine induced thyrotoxicosis	(7) Unresponsive to standard treatment (3) Agranulocytosis (1) Emergent preparation for thyroidectomy	Clinical improvement noted

Adali et al.	1	Gestational hyperthyroidism sec to molar pregnancy	Emergent preparation for thyroidectomy	Biochemical improvement with >80% decrease in FT3 and >75% decrease in FT4

Pasimeni et al.	1	Contrast induced hyperthyroidism	Unresponsive to methimazole	Clinical and biochemical improvement

Azezli et al.	1	Gestational hyperthyroidism sec to molar pregnancy	Preparation for emergent thyroidectomy	Clinical and biochemical improvement with 75.1% decrease in free t3 and 63.1% decrease in free t4

Erbil et al.	1	Gestational hyperthyroidism sec to molar pregnancy	Unresponsive to propylthiouracil	Biochemical improvement

Guvenc et al.	1	Toxic multinodular goiter	Agranulocytosis	Clinical and biochemical improvement

Ozbey et al.	4	Graves' disease	(1) Agranulocytosis (1) PTU induced vasculitis (1) Drug induced urticarial (1) Hepatotoxicity	Decrease in TT3 by about 40–78% and FT4 by >69%

Diamond et al.	3	Amiodarone induced thyrotoxicosis	Unresponsive to standard treatment	Clinical improvement in 2 patients Mild decrease in the FT4

Petry et al.	1	Graves' disease	Status after sleeve pneumonectomy	Clinical and biochemical improvement

Ozdemir et al.	1	Hyperthyroidism	Unresponsive to standard treatment	Clinical and biochemical improvement with 60% decrease in FT4 and 75% decrease in FT3

Segers et al.	5	Thyrotoxicosis	Thyrotoxicosis	Clinical improvement. Decrease in FT3 of 63.5% and FT4 by 57.8%

Ligtenberg et al.		Preparation for surgery	Preparation for surgery	Decrease in FT3 of 7% and 18% Decrease in FT4 of 0% and 33%

Samaras et al.	1	Amiodarone induced thyrotoxicosis	Unresponsive to standard treatment	Failure of treatment resulting in death of the patient Decrease in TT3 and TT4 noted after TPE with rebound increase in levels later

Aghini-Lombardi et al.	2	Amiodarone induced thyrotoxicosis	Adjunct to methimazole	Decrease in FT4 and FT3 Normalization of TT4 and TT3

De Rosa et al.	1	Hyperthyroidism	Agranulocytosis	Biochemical improvement with 51% decrease in FT3, 47% decrease in FT4, 60% decrease in TT3, and 53% decrease in TT4

Binimelis et al.	6	Levothyroxine intoxication	Cardiac and neurological symptoms	Clinical and biochemical improvement in 15 days

Jha et al.	1	Medicinal thyroid overdose	Medicinal thyroid overdose	Clinical and biochemical improvement with 43% decrease in TT4 and 68% decrease in TT3

Ashkar et al.	3	Hyperthyroidism	Severe hyperthyroidism	Clinical improvement in 2-3 days

Ft4: free thyroxine, Ft3: free triiodothyronine, TT4: total thyroxine, TT3: total triiodothyronine, and TPE: therapeutic plasma exchange.
